# Protease‐sensitive regions in amyloid light chains: what a common pattern of fragmentation across organs suggests about aggregation

**DOI:** 10.1111/febs.16182

**Published:** 2021-09-15

**Authors:** Giulia Mazzini, Stefano Ricagno, Serena Caminito, Paola Rognoni, Paolo Milani, Mario Nuvolone, Marco Basset, Andrea Foli, Rosaria Russo, Giampaolo Merlini, Giovanni Palladini, Francesca Lavatelli

**Affiliations:** ^1^ Amyloidosis Treatment and Research Center Fondazione IRCCS Policlinico San Matteo and Università Degli Studi di Pavia Italy; ^2^ Dipartimento di Bioscienze Università Degli Studi di Milano Italy; ^3^ Dipartimento di Fisiopatologia Medico‐Chirurgica e Dei Trapianti Università Degli Studi di Milano Italy

**Keywords:** amyloid fibrils, amyloidogenesis, immunoglobulin light chains, proteolysis, proteomics

## Abstract

Light‐chain (AL) amyloidosis is characterized by deposition of immunoglobulin light chains (LC) as fibrils in target organs. Alongside the full‐length protein, abundant LC fragments are always present in AL deposits. Herein, by combining gel‐based and mass spectrometry analyses, we identified and compared the fragmentation sites of amyloid LCs from multiple organs of an AL λ amyloidosis patient (AL‐55). The positions pinpointed here in kidney and subcutaneous fat, alongside those previously detected in heart of the same patient, were aligned and mapped on the LC’s dimeric and fibrillar states. All tissues contain fragmented LCs along with the full‐length protein; the fragment pattern is coincident across organs, although microheterogeneity exists. Multiple cleavage positions were detected; some are shared, whereas some are organ‐specific, likely due to a complex of proteases. Cleavage sites are concentrated in ‘proteolysis‐prone’ regions, common to all tissues. Several proteolytic sites are not accessible on native dimers, while they are compatible with fibrils. Overall, data suggest that the heterogeneous ensemble of LC fragments originates in tissues and is consistent with digestion of preformed fibrils, or with the hypothesis that initial proteolytic cleavage of the constant domain triggers the amyloidogenic potential of LCs, followed by subsequent proteolytic degradation. This work provides a unique set of molecular data on proteolysis from *ex vivo* amyloid, which allows discussing hypotheses on role and timing of proteolytic events occurring along amyloid formation and accumulation in AL patients.

## Graphical abstract

In immunoglobulin light‐chain amyloidosis (AL amyloidosis), fragments of the amyloidogenic light chain (LC) are always present in fibrillar deposits. We compared the fragmentation sites of amyloid LC from multiple organs of a patient. The fragments pattern coincides across tissues; multiple cleavage sites were concentrated in ‘proteolysis‐prone’ regions of the sequence, common to all tissues. This unique set of molecular data on *ex vivo* amyloid allows discussing hypotheses on role/timing of proteolysis in AL.

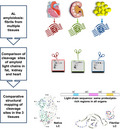

**Abbreviations**
2D‐PAGE: two‐dimensional polyacrylamide gel electrophoresis
ACN: acetonitrile
AL amyloidosis: immunoglobulin light‐chain amyloidosis
CL: light chain’s constant domain
Cryo‐EM: cryo‐electron microscopy
DTT: dithiothreitol
EA: ethanolamine
FA: formic acid
HCD: higher energy collisional dissociation
IEF: isoelectrofocusing
LCs: immunoglobulin light chains (note, LC in the standard hyphenated abbreviation LC-MS/MS indicates liquid chromatography)
PTM: post-translational modifications
VL: light chain's variable domain

## Introduction

The term amyloidosis indicates a group of diseases whose pathognomonic feature is the presence of extracellular protein aggregates with a specific cross β‐sheet fibrillar structure [1, 2, 3]. Approximately 40 distinct autologous proteins are currently known precursors of localized or systemic amyloid deposits in humans [3]. Among the systemic forms, light‐chain amyloidosis (AL amyloidosis) is the most frequent disorder in industrialized countries, and it is caused by widespread deposition of misfolding‐prone immunoglobulin free light chains (LCs), produced by a plasma cell clone in the bone marrow and circulating in the bloodstream [1, 4]. Light chains are ˜215 residues long proteins, whose primary sequence includes a variable and a constant region, which fold into two distinct domains [indicated as V domain, or light chain’s variable domain (VL), and C domain, or light chain’s constant domain (CL)] in the native protein [5, 6]. The combination of germline gene recombination and somatic hypermutation, occurring in the immunoglobulin genes, translates into high variability among LCs at the level of VL, so that the sequence of each patient’s monoclonal protein is virtually unique [7].

Fibril deposition is consequent to loss of the precursor’s native conformation, but the molecular events associated with LC fibrillogenesis *in vivo* and the tissue mechanisms counteracting the presence of amyloid fibrils are scarcely elucidated. The structures of *ex vivo* amyloid fibrils recently resolved using cryo‐electron microscopy (cryo‐EM) [8, 9, 10] show that the rigid fibrillar core is composed of the variable region of the LC and that the conversion from the native to the fibrillar form is associated with complete unfolding of VL [8, 9].

A prominent aspect in *ex vivo* AL fibrils is the fact that, alongside the full‐length monoclonal protein, LC fragments are always present and account for a large fraction of all the deposited species. The major fragments contain the complete VL and CL segments of variable length [11, 12, 13]. Several reports have indeed shown that fragments containing only portions of CL are also present in tissue fibrils, sometimes representing the major fibril component [11, 14, 15, 16].

To date, the origin of LC fragmentation is explained by two alternative working hypotheses: One postulates that LC cleavage near the joining region, with release of the amyloidogenic VL domain, triggers amyloidogenesis [17, 18, 19]; conversely, the other views the fragments as the result of postdeposition digestion of preformed fibrils [11, 15, 20, 21].

We recently implemented a mass spectrometry (MS)‐based approach to identify the *termini* of the LC fragments in tissue fibrils from the heart of two patients with AL λ amyloidosis [15]. The data showed that the cleavage sites share the feature of being located in poorly structured regions of the aggregates, suggesting that the proteolytic events observed in natural cardiac fibrils largely reflect postdeposition remodeling [15]. These considerations, however, are not intrinsically in contrast with the hypothesis that one or few proteolytic cuts on native LCs at specific positions destabilize the LC structure and trigger fibril formation, followed by recruitment of both truncated and full‐length LCs into fibrils, which are then further degraded.

An interesting additional observation previously reported [11] is the fact that, in individual patients with multiorgan involvement, the pattern of fragments is similar across tissues. These seminal biochemical findings have now also a morphological parallel: In fact, ultrastructural analyses recently showed that fibrils within a tissue can be polymorphic, but the distribution of polymorphs is similar in different organs [22, 23]. The existence of similar fragments across organs prompts specific questions in terms of proteolysis timing. On one hand, in fact, it may suggest a common origin of fragments from blood before deposition; on the other side, however, the structural identity of fibrils would translate into exposure of the same protease‐sensitive regions across organs, resulting in similar degradation. These open questions underline the need to precisely define the cleavage sites of amyloid LCs across tissues, in order to assess whether they differ between anatomical sites with distinct physiology and microenvironment and to hypothesize involved proteases. This is important missing information in the perspective of understanding the events that generate the LC proteoforms present in mature amyloid fibrils.

The objective of this study was to compare the fragmentation sites of amyloid light chains in multiple tissues of an affected individual and to analyze the accessibility of these sites on the native and fibrillar LC structures. To this aim, we combined high‐resolution gel‐based studies with LC‐MS/MS‐based terminomics analysis, for characterizing the N‐ and C‐terminal residues of fragments in fibrils from kidney and subcutaneous white fat from a patient (AL‐55), deceased due to AL λ amyloidosis, and for comparing the cleavage sites in these two organs with the previously reported proteolytic pattern in heart [15].

## Results

### 2D‐PAGE analysis: different tissues contain analogous populations of fragments

**Fig. 1:**
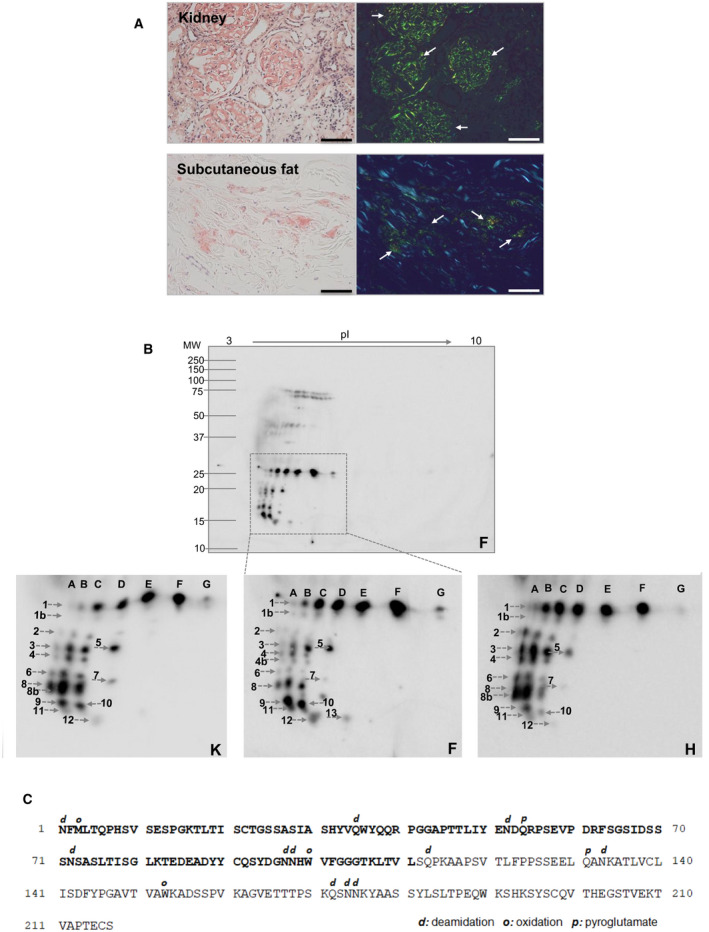
Amyloid LCs fibrils from tissues of patient AL‐55. (A) Congo red‐stained amyloid deposits in kidney and subcutaneous fat, visualized under nonpolarized (left panels) and polarized light (right panels). Amyloid deposits in kidney glomeruli and in adipose tissue interstitium show the pathognomonic apple‐green birefringence upon light polarization (arrows). Scale bar: 100 μm. (B) Western blotting analysis of amyloid light chains, enriched from adipose tissue, kidney, and heart. The fibrillar LCs were solubilized in IEF buffer, separated by 2D‐PAGE, and detected using a polyclonal anti‐λ LC antibody. Top panel: representative western blot referring to the entire 2D gel from adipose tissue. The region where monomeric full‐length LCs and fragments migrate is boxed (MW 25–10 kDa). Bottom panels: enlarged insets from the western blots of the three tissues (F: subcutaneous fat; K: kidney; H: heart), corresponding to the boxed region in the top panel. Matched pI isoforms of the full‐length LC across the three panels are labeled with the same letters; matched trains of fragments spots are labeled with the same numbers. (C) PTM detected in amyloid LCs from adipose tissue, indicated above each modified amino acid (d: deamidation; o: oxidation; p: pyroglutamate). Abbreviations: F: subcutaneous fat; K: kidney; H: heart; pI: isoelectric point; MW molecular weight.

The presence of amyloid fibrils in tissues was assessed by Congo red staining. The results of the histology analysis of kidney and subcutaneous fat are reported in Fig. 1A (histology data referring to the heart of the same patient had been previously shown [8]). Fibrils were typed as AL λ by immunoelectron microscopy. Kidney and fat tissue were used as sources of amyloid fibrils for the proteomic characterization. Two‐dimensional western blot analyses of previously described heart fibrils [15] were also run in parallel herein, in order to compare the population of spots from the three tissues. After repeated cycles of tissue homogenization in Tris‐EDTA buffer, the remaining tissue pellets, enriched in amyloid fibrils, were solubilized and subjected to 2D‐PAGE separation and anti‐λ LCs immunoblotting.

The western blots corresponding to the distinct organs are shown in Fig. 1B. Top figure shows the uncropped 2D WB from adipose tissue, representative of the pattern seen also in the other analyzed samples. The insets in bottom Fig. 1B display, side‐by‐side, the region of gels containing LCs in monomeric form in kidney, fat, and heart, respectively.

Corresponding spots, or ‘trains’ of spots, are labeled with the same numbers and letters across the panels. As previously reported [8, 12, 15], both the full‐length LC and the fragments are visible as horizontal trains of spots, referable to the presence of charge modification due to post‐translational modifications (PTMs), as described later. Inspection of the 2D WB data shows that the populations of fragments in the various tissues are strikingly superimposable in terms of MW and *pI* distribution of the fragments. The full‐length LC, with computed MW 23.3 kDa and computed *pI* 5.50, corresponds to the train of spots labeled as 1 (spots labeled from A to G). At least 13 distinct LC fragments can be discerned (numbered from 1b to 13) in each of the three samples; the relative abundance of each fragment, however, varies considerably across tissues (Fig. 1B). We previously observed that sequence coverage from in‐gel digested spots is suboptimal and does not allow reliable characterization of the fragment’s *termini* [15]; in addition, several spots contain a microheterogeneous mixture of fragments. These considerations prompted us to skip the MS characterization of the 2D‐PAGE spots and to perform instead an in‐solution LC‐MS/MS‐based definition of the cleavage sites, as reported later.

The presence of trains of spots with different *pI* reflects the fact that the amyloid LCs contain PTMs that modify their charge. Analysis of the gel spots was not diriment in defining which modifications differentiate the proteoforms, as previously observed [12]. We therefore investigated this aspect using LC‐MS/MS data and performed a targeted bioinformatic analysis to verify the presence of PTMs that change protein charge. Given the fact that the distribution of charge isoforms is analogous in the various tissues, we performed the PTM study only on the adipose tissue dataset, due to the particular purity of the fibrils from this site. The results are reported in Fig. 1C, in which the detected modifications are indicated over the corresponding amino acid residue on AL‐55 sequence. Partial oxidation of the only Met residue (Met 3) and of Trp 100 and Trp 153 was present. In addition, deamidation was confirmed to be a prominent phenomenon in amyloid LCs. In particular, all the asparagine residues of the sequence showed partial deamidation, as well as multiple glutamines (at positions 35 in the V region, and 113 and 172 in the C region). Deamidation possibly explains the appearance of several *pI* isoforms on 2D‐PAGE. Whereas asparagine deamidation can occur during sample preparation, deamidation of glutamines occurs at a much slower rate and is usually considered an *in vivo* process typical of long‐lived proteins [24, 25]. In addition, the extent of LC deamidation is higher than what observed in other proteins in the sample such as β‐actin (data not shown), likely indicating amyloid deamidation *in vivo*, on long‐lived amyloid fibrils that may persist for years in the body.

Notably, glutamines 54 and 131, at which levels truncation of the chain has been evidenced by the terminomics study, are also modified by conversion to pyroglutamate. This modification, in fact, occurs on glutamines located at the N *terminus* of proteins. No evidence of the other modifications explored (namely acetylation, phosphorylation, nitrosylation, methylation, or ubiquitination) was detected.

### Investigation of the cleavage sites of light‐chain fragments across tissues

Taking advantage of the recently implemented LC‐MS/MS‐based approach for assessing the LCs’ cleavage sites in *ex vivo* AL fibrils [15], we have herein characterized the C‐ and N‐terminal residues of the LC fragments in amyloid deposits from adipose tissue and kidney. The analogous characterization in relation to the heart of AL‐55 patient had been performed previously, and the reported data were used in the present study for comparative purposes [15].

As observed in the heart sample, AL‐55 was identified as one of the most abundant proteins also in kidney and fat, with complete MS/MS coverage of the amino acid sequence in both tissues. As previously reported in the case of the heart, other proteins were also identified in the tissue pellets with ≥ 2 unique peptides (1184 in the kidney and 353 in adipose tissue). Compared to heart and fat, the relative abundance of amyloid LCs in kidney with respect to the other tissue proteins was slightly lower, reflecting the uniquely glomerular localization of the amyloid deposits, which occupy a smaller fraction of the total tissue volume. The fewer number of cleavage sites found in kidney extracts likely reflects the lower amount of aggregates present in the tissue. The additional tissue proteins, overall, are not relevant for this study and will not be considered in the following discussion.

**Fig. 2:**
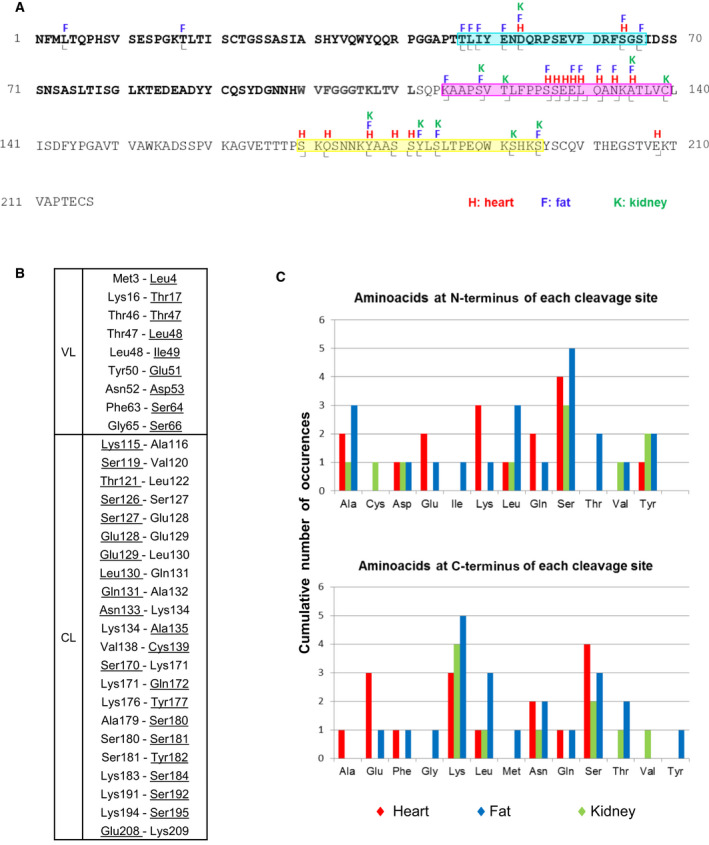
Mass spectrometry identification of the N‐ and C‐terminal amino acids of the amyloid LC fragments. (A) Amino acids identified as derivatized on the α amino group (N *termini* of the corresponding fragments) are indicated the by symbol └ below the sequence of AL‐55. Residues labeled on the α carboxylic group (C *termini* of the corresponding fragments) are indicated by the symbol ˩ below the sequence of AL‐55. The variable region of AL‐55 (aa 1–99) is indicated in bold black; the joining region (aa 100–111) is in bold gray. The colored letters above the sequence of AL‐55 indicate the tissue in which each labeled amino acid was detected (red H: heart; blue F: fat; green K: kidney. This color code is uniformly used in all panels of this figure). The N‐ and C *termini* of LC fragments from the heart of AL‐55 were identified in a previous study [15] and are reported here for comparison purposes. The three proteolysis‐rich regions are highlighted using the same colors as in Fig. 3A. (B) Cleaved peptide bonds, based on the fact that the amino acid at the N‐ or C‐terminal side of the bond was identified as labeled (corresponding, respectively, to the C‐terminal amino acid or to the N‐terminal amino acid of the corresponding fragment). The two amino acids involved in each bond are indicated; the labeled residues are underlined. (C) Cumulative occurrence of different amino acid residues at the N‐ or C‐terminal side of the cleaved bonds in AL‐55 LC, divided by organ in which cleavage was detected.

**Fig. 3:**
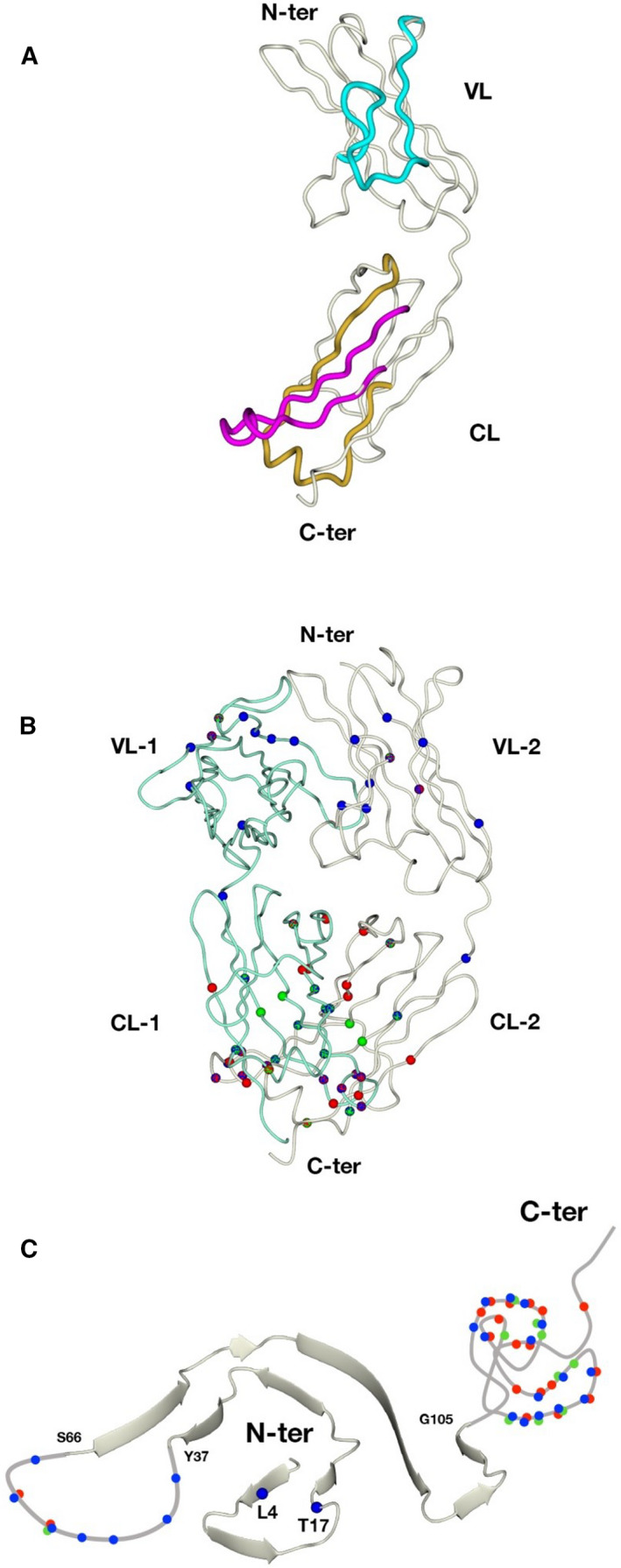
Mapping of cleavage sites on native and fibrillar structures. (A) The three ‘cleavage‐prone’ segments (47–66 in VL, 115–139, and 170–195 in CL) are mapped on the structure of native JTO LC monomer. The colors of the three segments correspond to those used in Fig. 2A. Figures are generated using CCP4mg [39]. The derivatized amino acids identified in adipose tissue (blue dots) and kidney (green dots) are mapped on the structures of (B) native JTO light‐chain dimer (pdb: 6MG4) and (c) fibrillar AL‐55 (pdb: 6HUD). The cleavage sites previously identified in heart (red dots) [15] are also shown for comparison. Dots with mixed colors indicate positions shared by more than one tissue.

In adipose tissue, excluding the canonical N *terminus* of the sequence, 14 internal amino acids were confidently identified as dimethylated, indicating that they contain a free amino group on the α carbon and are therefore the N *termini* of corresponding LC fragments (Fig. 2A, residues labeled with letter ‘F’ and symbol 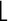, and Fig. 2B). In addition, 7 internal residues were found to be derivatized with ethanolamine (EA) on the α carboxyl group, indicating that they are the C *termini* of corresponding LC fragments (Fig. 2A, residues labeled with letter ‘F’ and symbol 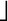, and Fig. 2B). The position of all cleavage sites found in adipose tissue are colored in blue and shown in the primary sequence, on the native structure of a dimeric LCs and on the structure of fibrillar AL‐55 in Fig. 3.

Eight of the 14 N‐terminally labeled positions in fat are coincident with positions identified also in the heart and/or kidney (Fig. 2A). The majority of the remaining N‐terminally labeled cleavage sites (Thr 47, Leu 48, Ile 49, Glu 51, Ser 66, Tyr 182, Ser 184, and Ser 195), despite not coinciding with those detected in the other organs, are however located in specific regions in AL‐55 sequence, previously demonstrated to be ‘cleavage‐sensitive’ also in the heart (in particular regions 47–66 and 170–195) [15] (Fig. 2A). Two novel N *termini* (Leu 4 and Thr 17) are unique for adipose tissue and are localized in the proximal portion of the V domain. All the C‐terminally labeled residues identified in fat are concentrated in a restricted region of the sequence that spans residues 115–139, where fragmentation sites were concentrated in heart fibrils (Fig. 2A). Five of these 7 C‐terminally labeled residues coincide with those found in the heart; an additional one is shared with kidney (Fig. 2A).

In fibrils from kidney, 8 amino acids were dimethylated on the α amino group (Fig. 2A). Six of these are in common with heart and/or fat (Fig. 2A); the remaining 2 (Cys 139 and Ser 192) fall within previously mentioned ‘cleavage‐sensitive’ segments. Two amino acids were labeled on the α carboxyl group. One position (Ser 119) is labeled also in fat, and both Ser 119 and Thr 121 fall within the ‘cleavage‐sensitive’ region 115–139 (Figs 2A and 3A).

We then considered each identified fragmentation point in the LC sequence, regardless of whether labeled at the amino or the carboxyl side, and we computed the frequency with which different amino acids occurred at either side of the bond. The results of this analysis are shown in Fig. 2C. Overall, several distinct amino acids are found at both sides of the breakage points; however, predominance of lysine residues at the C‐side and of serine residues at the N‐side was observed.

The structure of cardiac amyloid fibrils from patient AL‐55, previously obtained, and that of JTO LC [19], whose primary sequence is highly homologous to that of AL‐55, were used as surrogates to map the LC cleavage sites identified in fat and kidney (Fig. 3B,C).

Overall, a global look at the proteomic data obtained for the three organs (Figs 2 and 3) shows that (a) several labeled positions in each organ are shared by at least another organ. Three fragmentation sites are identical in all the tested tissues (position 53 in the VL, 135 and 177 in CL); (b) the vast majority of all fragmentation sites in the three organs are concentrated in three specific ‘fragmentation‐rich’ segments of the LC (Figs 2A and 3A): residues 47–66 (VL domain), 115–139 (proximal part of the CL domain), and 170–195 (distal part of the CL domain). In the structure of AL‐55 heart fibrils previously determined by cryo‐EM [15] (Fig. 2B), these regions are not structured and therefore are plausible targets of proteolytic degradation. Exceptions to this observation are the two residues Leu 4 e Thr 17 identified as labeled only in fat, which fall in the N‐terminal segment of the V region. This zone, in heart fibrils, belongs to the structured core [8, 15]. This observation may be due to partially aggregated species copurified with amyloid deposits.

Notably, the microheterogeneity in fragmentation sites across organs is not in contrast with the substantial coincidence of 2D‐PAGE spots. In fact, gel separation of fragments with very close MW and *pI* is not complete, and the same protein spot can contain multiple proteoforms differing for a minimal number of amino acids. Indeed, as mentioned above, the fact that each 2D gel protein spot contains a microheterogeneous ensemble of proteoforms had already been reported [15].

## Discussion

In this work, we performed a combined gel‐based and gel‐free proteomic characterization of the amyloid LCs extracted from adipose tissue and kidney from patient AL‐55, deceased due to AL λ amyloidosis. The cleavage sites in cardiac LC fibrils from AL‐55 had been previously defined and mapped on the native and fibrillar LC structures [15] and have been considered herein for comparison purposes. Overall, all the cleavage sites that had been detected in cardiac fibrils shared the feature of being located in nonstructured regions of the fibrillar aggregates: This had led to hypothesize that most of the observed cleavage events that generate the fragment visible in mature heart LC fibrils occur after deposition, on proteolysis‐sensitive structural segments [15].

The present analyses extend the amyloid LC characterization to multiple affected tissues, with the aim to investigate whether the fragmentation mechanisms are similar across organs with distinct physiology and extracellular environments. This study provides important novel information for elucidating the mechanisms of LC processing. In particular, our data show that (a) all tissue deposits contain fragmented LCs along with the full‐length protein; (b) by gel analysis, the pattern of LC fragments is substantially coincident across tissues, although the relative abundance of the various proteoforms varies slightly from site to site; (c) multiple cleavage positions can be detected by LC‐MS/MS in all tissues, consistently with the presence of many fragments. Some of the cleavage positions are shared by various tissues, whereas some are organ‐specific; (d) the cleavage sites are not uniformly distributed along the AL‐55 LC sequence, but they are instead concentrated in specific ‘cleavage‐prone’ regions, which appear to be conserved in all the three tested tissues (Fig. 3A).

The fact that all tissues contain a mixture of full‐length LC and fragments is a known phenomenon; in addition, previous analyses based on custom antibodies directed against distinct regions of the light chains had shown that the fragments of each patient are similar across organs [11]. From a methodological point of view, the 2D electrophoresis used herein provides increased resolution compared to one‐dimensional SDS/PAGE, because it separates also proteoforms with the same MW but different charge. Indeed, our 2D‐PAGE data confirm and further reinforce the concept that the overall proteolytic pattern is very similar in fat, kidney, and heart.

The LC‐MS/MS‐based data here reported provide additional crucial information: the precise mapping of LC fragmentation sites, an aspect that was hard to define using traditional gel electrophoresis analysis. Our high‐resolution data indicate that several fragmentation points are identical across tissues and a large proportion of the overall detected sites are shared by at least two of the tested organs; importantly, they also suggest that microheterogeneity exists in fragmentation positions.

The presence of similar LC fragments in different tissues could be explained by two alternative hypotheses. Such fragments may have an identical origin, that is, proteolysis occurs in blood and proteolysed LCs circulate before reaching different organs. An opposite possibility is that LC fragments originate locally in different tissues, from digestion of the same regions/positions. Our data are more consistent with the second hypothesis, for several reasons. First, although the fragmentation sites are concentrated in the same regions of AL‐55 across different tissues (residue 47–66 in VL, aa 115–139, and aa 170–195 in CL), the population of LC fragments in mature AL fibrils is complex and rather diversified from tissue to tissue. It is unlikely that such complex pattern may be so precisely reproduced across tissues from aggregation of common circulating proteolysed proteoforms. Secondly, a relevant number of proteolytic sites are not accessible on the native circulating LC dimers while are fully compatible with aggregated LCs. Finally, the full‐length LC is an important constituent of the fibrils, as well as fragments containing portions of the C domain, indicating that the nonfragmented LC participates in the formation of amyloid fibrils.

It is noteworthy that the presence of LC fragments in AL patients’ urines is a well‐known phenomenon, while the existence of significant populations of fragments in blood has not yet been consistently demonstrated [12, 26, 27, 28]. Nevertheless, MS analyses evidenced differences between urinary and deposited fragments, which may thus be generated through distinct mechanisms [29]. Urinary fragments may be due to bacterial proteases or other proteases leaked during proteinuria that is often present in AL amyloidosis, or even during transit across the nephron and trafficking/degradation in tubular cells. The presence of urea may contribute to unfold the LCs, facilitating digestion especially at the level of the more flexible variable domain of amyloidogenic LCs. Fragmentation of proteins in urines is indeed not uncommon [30]. Therefore, the heterogeneous ensemble of LC fragments present in mature amyloid is overall more likely to derive from *in loco* processing at the level of affected tissues. The above‐mentioned microheterogeneity in cleavage points across organs likely reflects the action of different proteases in any given tissue. The overall pattern of proteolysis is thus consistent with the combined action of a complex mixtures of proteolytic enzymes, as previously suggested in relation to heart fibrils [15]. Analysis of amino acids at each side of the proteolysed peptide bonds (Fig. 2C) does not suggest a clear prevalence of specific enzymes, although a marginal predominance of lysines at the C‐side was present, possibly related to the action of proteases with trypsin‐like specificity. Taken together, our data and the above considerations suggest that proteolysis of LCs most likely occurs once these proteins are localized at the target organs rather than when circulating in blood.

Even though the present data only provide a snapshot of the end products of LC aggregation and do not carry any direct evidence on the mechanisms of amyloid formation, we can nevertheless use the information on the proteolytic pruning in *ex vivo* fibrils to cast hypotheses on the fibrillogenesis pathways. Indeed, models should be compatible with the features of natural aggregates, here characterized in detail.

VL domains are typically more amyloidogenic than the respective full‐length LC [17, 18, 19, 31, 32], and it has been proposed that a proteolytic cleavage in the linker region is necessary to unleash the amyloidogenic potential of VL. In this context, VL domain is viewed as the real responsible species of LC aggregation. Such hypothesis is not in agreement with our data (no cleavage sites have been detected in the joining region). Although possible, we deem it unlikely that such an aggregation‐prone species (i.e., isolated VL domain) is present only in such low amount that cannot be detected in our experiments.

A first model compatible with our data is represented by the hypothesis that no proteolysis occurs before aggregation. In the structure of LC fibrils, CL domains are invisible and thus disordered [8, 9, 10]; however, were CL domains totally unfolded in mature fibrils, proteolysis would be homogenous along the CL sequence, while we observed a concentration of cleavage points in ‘proteolysis‐rich’ segments (Fig. 3A). To explain the specific proteolysis‐rich segments observed on CL domains, we should therefore assume that such domains may not be natively structured, but anyway folded in a specific conformation, in which except the two proteolysis‐rich segments the rest of the CL domain is protected from protease attack. Protection from the proteolytic attack may otherwise be conferred by association of specific CL segments with other molecules, such as the extracellular chaperones that are known ubiquitous components of the amyloid deposits *in vivo*.

However, another alternative explanation is also possible, in which the final proteolytic pattern is generated by sequential events occurring at different time points on distinct LC conformational states. The general view is that CL domains exert a stabilizing effect on VL domains [19, 31, 33, 34]. Nevertheless, recent data indicate that full‐length LCs with destabilized CL domains or nonoptimal interdomain interactions are highly amyloidogenic [31, 35, 36]. It is possible that initial proteolytic events on exposed loops (aa 120–131 and 181–190) destabilize the CL domain, which in turn renders the whole native LC more aggressively amyloidogenic. This may also help VL domains to rapidly unfold and misfold to form the mature fibrillar conformation, which is readily protected from proteolysis [8, 9]. Further sequential proteolysis of the CL domain may occur preferentially in the 115–139 and 170–195 regions because these regions are highly flexible and exposed due to the first proteolytic events. The CL proteolytic pattern is broadly conserved in three different tissues of AL‐55 patient and also in the heart of another patient (AL‐H7) [15], whose fibrils were formed by a LC belonging to a different germline. Such conservation may suggest a common chain of molecular events. As far as the proteolytic pattern of the VL domain is concerned, the proteolysis observed in the AL‐55 and AL‐H7 tissues is best viewed as postaggregation remodeling.

Along with the above considerations, some caveats shall be taken in account. First, although the proteolytic patterns are clearly well conserved, the profile of proteolysis has so far been analyzed only in four different tissues from two different patients (AL‐55 and in previously described AL‐H7 [15]); therefore, additional analyses may be necessary to generalize such considerations to the whole population of AL patients. This study does not provide quantitative information on each cleavage product; even in the WB analyses, the relative abundance of the fragments cannot be rigorously compared. In fact, as previously observed in the heart [15], the polyclonal primary antiserum preferentially recognizes the full‐length LC and fragments containing the constant domain, whereas fragments containing mostly the variable domain display a weaker signal. Of note, the mass spectrometry analyses, which represent the core of the study, were performed on light chains that had by no means been enriched using antibodies and therefore provide a snapshot that is not affected by the preferential reactivity for specific epitopes. In the future, complementing the descriptive data provided herein with quantitative assessment of fragment abundance will be instrumental to precisely define the importance of each cleavage site and to shed additional light on the mechanisms of fibrillogenesis *in vivo*. Finally, since amyloid distribution is not homogeneous in tissues and the three tested tissues differ in architecture and anatomical features, it may be possible that differences exist in the extraction performances and in the relative abundance between LC and remaining tissue proteins. For these reasons, we have mainly focused our attention on the pattern and distribution of cleavages sites in the various tissues, rather than specific proteolytic sites.

In summary, the present data, overall, provide a precise molecular description of natural AL amyloids *ex vivo* from different organs of an individual patient. Such molecular understanding allows to critically discuss available hypotheses on the specific proteolytic events occurring upon amyloid deposition and accumulation in AL patients.

## Materials and Methods

### Enrichment of amyloid fibrils from tissues of patient AL‐55

Amyloid fibrils were enriched from three distinct tissues (heart, kidney and subcutaneous abdominal fat) acquired during autopsy from patient AL‐55, affected by AL λ amyloidosis with clinical involvement of heart and kidney and positivity of fat for amyloid during the diagnostic workup. The clinical features of patient AL‐55 have been described in detail in previous publications [8]. Immediately after acquisition, unfixed tissue specimens were stored at −80 °C and kept frozen until use. The sequence of the monoclonal λ LC from patient AL‐55 derives from the IGLV6‐57 germline gene and has been previously deposited in GenBank with code MH670901 [8, 15]. The cryo‐EM structure of *ex vivo* fibrils extracted from the heart of patient AL‐55 was previously defined and has been deposited in Protein Data Bank with code 6HUD [8].

All the procedures for enrichment of fibrils for proteomics were performed on ice in presence of a cocktail of protease inhibitors (Complete, Roche, Basel, Switzerland), as previously described in detail [15]. Briefly, each tissue specimen (approximately 250 mg) was washed 5 times in cold PBS/protease inhibitors and then homogenized in Tris‐EDTA/protease inhibitors buffer (20 mm Tris, 140 mm NaCl, and 10 mm EDTA, pH 8.0) with a glass potter pestle. The sample was centrifuged at 3100 ***g*** after homogenization, and the supernatant was discarded to remove soluble tissue and blood proteins. The overall homogenization procedure in Tris‐EDTA was repeated 10 times, after which the remaining pellet was rinsed once with ultrapure water and then used for the proteomic analyses.

The patient had provided antemortem informed consent for acquisition and use of biological samples for research purposes. The research studies on amyloid fibrils from human autopsy samples were approved by the Ethical Committee of Fondazione IRCCS Policlinico San Matteo; research was performed in accordance with the Declaration of Helsinki.

### Two‐dimensional polyacrylamide gel electrophoresis (2D‐PAGE) and western blotting

For protein quantification, the protein pellet was incubated for 1 h with 8 M urea in order to dissolve protein aggregates, followed by centrifugation to remove nonsolubilized material. Protein samples were quantified using micro‐BCA assay (Thermo Fisher Scientific, Waltham, MA, USA). For 2D‐PAGE and western blotting, 100 μg of proteins were diluted with DeStreak buffer (GE Healthcare, Chicago, IL, USA) and 0.1 M dithiothreitol (DTT). Isoelectrofocusing (IEF) was performed using 11 cm 3–10 NL pH gradient strips (Bio‐Rad, Hercules, CA, USA); the second dimension was performed using precast 8%–16% polyacrylamide gradient gels (Bio‐Rad), under denaturing and reducing conditions. The detailed protocol for 2D‐PAGE has been previously described [8, 15]. For immunoblotting, a rabbit polyclonal antibody against human lambda light chains (Agilent Dako, Santa Clara, CA, USA) was employed [15].

### Labeling of the free N‐ and C‐ termini of the amyloid LC fragments

The procedures for covalently labeling the C‐ and N‐terminal residues of the LC proteoforms have been previously described in detail [15]. Briefly, after reduction of disulfide bonds and alkylation of cysteines, primary amines in the reconstituted samples were dimethylated using formaldehyde and NaBH_3_CN, resulting in covalent modification of the free primary amino groups, including those bound to the α carbon of N‐terminal amino acids. For labeling of the C‐terminal amino acids, charge‐reversal derivatization of the carboxyl groups (including those bound to the α carbon of C‐terminal amino acids) was obtained by amidation with EA, performed on the proteins previously subjected to amine protection (i.e., through the primary amine derivatization procedure mentioned above). Prior to LC‐MS/MS analysis, all samples (unlabeled protein extracts, samples subjected to amine dimethylation for study of the N *termini* and samples subjected to labeling of the carboxyl groups) were digested with trypsin (Sequencing Grade Modified Trypsin, Promega, Madison, WI, USA), as described [15].

### LC‐MS/MS analysis and database search

LC‐MS/MS analyses were performed on a Dionex Ultimate 3000 nano‐UHPLC RSLC system coupled to a Q Exactive Plus mass spectrometer (Thermo Fisher Scientific, Waltham, MA, USA) equipped with an EASY‐spray ion source (Thermo Fisher Scientific), as previously described in detail [15]. After washing on a trap column (PepMap100 C18, 0.3 x 5 mm, 5 µm, 100 Å, Thermo Fisher Scientific), peptides were separated on an analytical column (PepMap RSLC C18, 75 µm x 50 cm, 2 µm, 100 Å, Thermo Fisher Scientific), at a flow rate of 250 nl·min^−1^, using a linear gradient from 2% of acetonitrile (ACN)/formic acid (FA) 0.1%–35% in 61 min and from 35% to 95% in 12 min. Acquisition of mass spectra in 300–2000 m/z range was operated in positive ion mode. The 10 most intense precursor ions were selected for higher energy collisional fragmentation (HCD). Data were processed using the Sequest HT‐based search engine contained in the proteome discoverer software, version 2.0, from Thermo Scientific.

In a preliminary database search step, the MS/MS data were searched against the human proteome database (downloaded from UniProt in August 2019 and containing 74 190 entries) additioned of the sequence of AL‐55; full tryptic cleavage and carbamidomethylation of cysteines as fixed modification were specified. This step allowed creating a subdatabase (target database) containing the entries identified in the sample. The target database was used in the second step of spectra alignment, in which semitryptic cleavage and additional PTMs were indicated. Specifically, these modifications included the following: (a) in samples labeled as described above: dimethylation of lysines and N *terminus* (+28.031) and addition of EA on aspartate, glutamate, and C *terminus* (+43.042); (b) in unlabeled samples: oxidation of methionines and triptophans (+15.995), cyclization of N‐terminal glutamines (−17.027), deamidation of glutamines and asparagines (+0.984), acetylation on lysines and N *terminus* (+42.011), ubiquitination on lysines (by investigating the presence of a diglycine peptide bound to the ε‐amino group of lysines (Kε‐GG), +114.043), phosphorylation of serines, threonines, and tyrosines, (+79.966), nitrosylation of triptophans and tyrosines, (+44.985), methylation of aspartic acids, glutamic acids, histidines, lysines, arginines, serines, and threonines (+14.016). A maximum of 5 dynamic modifications per peptide were allowed. All spectral matches from peptide ions putatively labeled or containing PTMs were manually checked before being confirmed. The mass spectrometry proteomics data have been deposited to the Proteome Xchange Consortium via the PRIDE partner repository [37, 38] with the dataset identifier PXD025185. Other raw data will be shared upon request (contact Francesca Lavatelli, E‐mail: francesca.lavatelli@unipv.it).

## Conflict of interest

The authors declare that they have no conflicts of interest with the contents of this article.

## Author contribution

FL, GM, and SR conceptualization; FL, GM, SR, PR, and SC data acquisition and curation; FL, GM, and SC formal analysis; FL, SR, GM, and GP supervision; FL, SR, MN, and GP funding acquisition; FL, GM, SR, PR, and GP writing‐original draft; FL and GP project administration; FL, GM, SR, PR, PM, MN, MB, AF, RR, GM, and GP writing‐review and editing; GP and GM resources; PM, MB, AF, MN acquisition and clinical characterization of patients' samples; SR and RR structural mapping.

## Data Availability

The mass spectrometry proteomics data have been deposited to the Proteome Xchange Consortium via the PRIDE partner repository with the dataset identifier PXD025185. Other raw data will be shared upon request (contact Francesca Lavatelli, E‐mail: francesca.lavatelli@unipv.it).
